# Interrater reliability for the detection of cortical lesions on phase-sensitive inversion recovery magnetic resonance imaging in patients with multiple sclerosis

**DOI:** 10.1590/0100-3984.2022.0116

**Published:** 2023

**Authors:** Marco Aurelio Gralha de Caneda, Marjana Reis Lima Rizzo, Gabriela Furlin, Abraão Kupske, Bruna Bressan Valentini, Rafaela Fiss Ortiz, Camila Batista de Oliveira Silva, Maria Cecilia Aragon de Vecino

**Affiliations:** 1 Hospital Moinhos de Vento, Porto Alegre, RS, Brazil

**Keywords:** Multiple sclerosis/diagnostic imaging, Cerebral cortex/pathology, Magnetic resonance imaging/methods, Image processing, computer-assisted, Observer variation, Esclerose múltipla/diagnóstico por imagem, Córtex cerebral/patologia, Ressonância magnética/métodos, Processamento de imagem assistida por computador, Variações dependentes do observador

## Abstract

**Objective:**

To assess the reliability of phase-sensitive inversion recovery (PSIR) magnetic resonance imaging (MRI) and its accuracy for determining the topography of demyelinating cortical lesions in patients with multiple sclerosis (MS).

**Materials and Methods:**

This was a cross-sectional study conducted at a tertiary referral center for MS and other demyelinating disorders. We assessed the agreement among three raters for the detection and topographic classification of cortical lesions on fluid-attenuated inversion recovery (FLAIR) and PSIR sequences in patients with MS.

**Results:**

We recruited 71 patients with MS. The PSIR sequences detected 50% more lesions than did the FLAIR sequences. For detecting cortical lesions, the level of interrater agreement was satisfactory, with a mean free-response kappa (κ_FR_) coefficient of 0.60, whereas the mean κ_FR_ for the topographic reclassification of the lesions was 0.57. On PSIR sequences, the raters reclassified 366 lesions (20% of the lesions detected on FLAIR sequences), with excellent interrater agreement. There was a significant correlation between the total number of lesions detected on PSIR sequences and the Expanded Disability Status Scale score (ρ = 0.35; *p* < 0.001).

**Conclusion:**

It seems that PSIR sequences perform better than do FLAIR sequences, with clinically satisfactory interrater agreement, for the detection and topographic classification of cortical lesions. In our sample of patients with MS, the PSIR MRI findings were significantly associated with the disability status, which could influence decisions regarding the treatment of such patients.

## Abstract (pt)

**Objetivo:**

Avaliar a confiabilidade da sequência PSIR e sua precisão no diagnóstico topográfico de lesões corticais desmielinizantes em pacientes com esclerose múltipla (EM).

**Materiais e Métodos:**

Estudo transversal realizado em centro de referência terciário para EM e distúrbios desmielinizantes. Avaliamos a concordância entre três avaliadores na identificação e classificação topográfica de lesões corticais na ressonância magnética de pacientes com EM, utilizando as sequências FLAIR e PSIR.

**Resultados:**

Foram incluídos 71 pacientes com EM. Em PSIR detectou-se 1,5× mais lesões do que em FLAIR, com concordância satisfatória entre examinadores na identificação de lesões corticais, com coeficiente kappa de resposta livre (κ_FR_) = 0,60, e na reclassificação topográfica das lesões, com κ_FR_ médio = 0,57. Os avaliadores reclassificaram 366 lesões em PSIR (20% das lesões detectadas em FLAIR), com excelente concordância. Houve correlação significativa do total de lesões detectadas em PSIR e o escore da escala de incapacidade EDSS (ρ = 0,35; *p* < 0,001).

**Conclusão:**

PSIR mostrou-se superior na detecção de lesões corticais e na classificação topográfica destas em comparação ao FLAIR, com concordâncias entre examinadores clinicamente satisfatórias. A associação significativa entre o número de lesões corticais em PSIR e o grau de incapacidade dos pacientes pode influenciar em decisões terapêuticas.

## INTRODUCTION

Multiple sclerosis (MS) is a chronic, inflammatory, demyelinating degenerative disease of the central nervous system (CNS) and has a markedly heterogeneous clinical course^(1)^. Although the etiology of MS is unknown, the interaction between genetic and environmental factors probably plays a relevant role in its onset^(1,2)^. In terms of the etiopathogenesis, autoreactive inflammatory cells cross the blood-brain barrier and induce CNS damage, leading to the formation of demyelinating lesions^(1-4)^.

Historically, MS was considered as a disease of the white matter. However, various magnetic resonance imaging (MRI) studies of patients with MS have consistently shown that demyelinating lesions are common in the gray matter of cortical and deep brain structures of such patients and can be extensive^(5-9)^. Accurate assessment of gray matter damage has contributed significantly to the interpretation of the association that cortical lesions have with clinical and cognitive outcomes in MS^(8,9)^. Depending on their location, cortical lesions can be classified as leukocortical (type I), intracortical (type II), or subpial (subdivided into types III and IV). These diverse topographies suggest that the onset and growth of cortical lesions occur through various mechanisms, and their distinct locations probably account for the heterogeneous course of MS^(3,4,9,10)^. Type I cortical lesions can be confused with juxtacortical lesions, which are quite common, being found in 50% of patients with MS in autopsy studies^(4,11)^.

For the diagnosis and follow-up of patients with MS, it is essential to perform MRI examination of the CNS. That examination can provide information regarding the activity and progression of the disease, which are used as criteria for treatment failure, as well as being useful in screening for complications of MS treatment^(12,13)^. The standard MRI protocol for MS includes volumetric fluid-attenuated inversion recovery (FLAIR), T1-weighted, gadolinium contrast-enhanced T1-weighted, and T2-weighted sequences. Those conventional sequences present low sensitivity for the detection of cortical lesions, even if the images are acquired in high-power scanners. In contrast, non-conventional pulse sequences, such as those based on phase-sensitive inversion recovery (PSIR) or double inversion recovery (DIR), substantially improve the sensitivity for the detection of cortical lesions^(7,8)^.

A PSIR sequence is T1-weighted and presents a marked contrast between white and gray matter. It has a higher signal-to-noise ratio than does a DIR sequence, with faster acquisition times, resulting in images with higher resolution. On a PSIR sequence, the distinct boundary between the cortical strip and adjacent white matter provides a more accurate differentiation between cortical lesions, juxtacortical lesions, and white matter lesions^(14-16)^. Lesions identified as fully localized in gray matter on other sequences, such as FLAIR and DIR sequences, might in fact be juxtacortical lesions or be totally located in the white matter. The intrinsically low signal-to-noise ratio of DIR sequences, which are widely used in the detection of cortical lesions, does not allow increases in resolution beyond that achieved during the long periods required for the acquisition of clinically acceptable scans. In contrast, PSIR sequences can provide higher image resolution within shorter, clinically feasible periods, the typical scan time being approximately 11 min^(15,16)^. Some studies have demonstrated that PSIR sequences have higher accuracy than do FLAIR sequences and are 3-4 times more sensitive than are DIR sequences for the detection of cortical lesions^(12,16,17)^. In addition, the PSIR findings have been shown to correlate significantly with physical and cognitive function in patients with MS; therefore, given the clinical relevance of cortical damage in MS, the PSIR findings could have important diagnostic and prognostic implications^(11,18)^.

The most recent revision of the diagnostic criteria for MS incorporated the presence of cortical lesions as a discriminating criterion for the spatial dissemination of the disease^(19)^. This new parameter made it crucial to assess the accuracy of recently developed MRI sequences used in order to detect these lesions. Therefore, we assessed the performance of PSIR sequences and their accuracy for determining the topography of demyelinating cortical lesions in patients with MS.

## MATERIALS AND METHODS

This was a cross-sectional study of consecutive patients with MS who underwent MRI of the CNS within a 12-month period, based on the recommendations put forth in the Standards for Reporting of Diagnostic Accuracy Studies^(20)^ and in the Guidelines for Reporting Reliability and Agreement Studies^(21)^. The study was approved by the local research ethics committee, and all participating patients gave written informed consent.

The MRI sequences were acquired in a 3.0-T scanner (Spectra; Siemens Medical Systems, Erlangen, Germany), with a 32-channel head/neck coil. The image acquisition protocol consisted of the following: unenhanced T1-weighted turbo spin-echo, 3D T1-weighted gradient-echo, and 3D FLAIR sampling perfection with application-optimized contrasts using different flip angle evolution sequences, all in the sagittal plane; T2-weighted turbo spin-echo and diffusion-weighted echo-planar imaging sequences, both in the axial plane; intravenous infusion of gadolinium-based contrast (Gadovist; Bayer Schering Pharma AG, Berlin, Germany), with dosing according to patient body weight; and acquisition of contrast-enhanced axial susceptibility-weighted imaging and sagittal 3D T1-weighted gradient-echo sequences, together with axial T1-weighted fast spin-echo magnetization transfer contrast and axial PSIR sequences. The main sequences of interest were 3D FLAIR and PSIR. The following parameters were used for 3D FLAIR: field of view, 260 mm; repetition time/echo time, 5,000/395 ms; matrix, 256 × 256; voxel size, 0.5 × 0.4 × 1.0 mm; and acquisition time, 5 min 15 s. The parameters used for PSIR were field of view, 220 mm; repetition time/echo time, 2,600/10 ms; matrix, 195 × 320; voxel size, 0.4 × 0.4 × 3.0 mm; and acquisition time, 3 min 47 s. Cortical lesions were identified on the basis of recommendations in the literature^(12,15,17)^. Two neuroradiology interns, each with one year of experience, designated rater 1 and rater 2, respectively, evaluated images obtained from the picture archiving and communication system, and their findings were compared with those of a senior rater with 14 years of experience (six dedicated to patients with MS), designated rater 3, who evaluated the same images.

The Expanded Disability Status Scale (EDSS) score, duration of disease, MS phenotype, sex, and age were extracted from patient electronic medical records. All of the raters were blinded to the patient clinical data and to the findings of their peers. Each rater identified and counted the juxtacortical and cortical lesions in the supratentorial and infratentorial regions, first on the FLAIR sequences and then on the PSIR sequences, which were considered the standard sequences to define the topographic classification of the cortical lesions.

### Statistical analysis

The findings of each rater are presented as the means and standard deviations (SDs) of the lesion counts. After applying the Shapiro-Wilk test to verify the distribution of the variables, we estimated the correlation coefficients for the associations that the numbers of juxtacortical and cortical lesions had with the EDSS score, duration of disease, and patient age.

To calculate the agreement between the findings of the two inexperienced raters and those of rater 3, for the presence of lesions, their localization, and topographic classification, we used the free-response kappa (κ_FR_) statistic. That was chosen because the traditional assessment of interrater agreement can be misleading in situations in which only positive findings are computed, such as in imaging examinations. In such examinations, the lack of a specific number for negative observations (absence of lesions) limits the use of the classical kappa statistic. In many cases, the images must cover extensive areas of bulky organs. In addition, multiple abnormalities, such as MS plaques, make the measurement of agreement impractical or biased by a free-response paradigm, leading to underestimation of kappa coefficients^(22)^. With the κ_FR_ statistic, the agreement does not depend on unknown/negative data and can be estimated from positive results alone, which is particularly useful in imaging studies^(22)^. The classification of the κ_FR_ follows the standard Fleiss ratings employed for the kappa coefficient^(23)^, categorizing agreement as poor (< 0.40), satisfactory (0.40 < κ_FR_ < 0.75), or excellent (> 0.75). Intraclass correlation coefficients (ICCs), with two-way random effects for agreement between raters, which is appropriate for examiners with different levels of experience^(24,25)^, were calculated for the total lesion counts on FLAIR and PSIR sequences, as well as for the reclassification of cortical lesions (defined as any change in their topographical classification on PSIR sequences).

The concordance between FLAIR and PSIR images for the same rater (intrarater agreement) was assessed with a Bland-Altman plot, which represents the repeatability between different methods, analyzing the averages of the differences in the number of lesions found by one rater. To be clinically acceptable, 95% of the differences between the methods must remain within a range of the mean of the differences ± 1.96 SD, which would indicate the potential for interchangeability between the methods^(26)^. Therefore, the results are expressed as means and SDs, with 95% confidence intervals (CIs). To exclude a proportion bias, we performed regression analysis of the differences between the methods. On the basis of data in the literature, we established *a priori* a limit of seven lesions as a clinically relevant and acceptable difference between the sequences^(27-29)^. The sample size was calculated in order to provide clinically satisfactory coefficients of agreement, at a significance level of *p* < 0.05 and a power of 0.80. For the correlation analysis, Spearman’s rho (ρ) was calculated. The statistical analysis was performed using the Stata software, version 14.0 (Stata Corp LP, College Station, TX, USA).

## RESULTS

**Figure 1:**
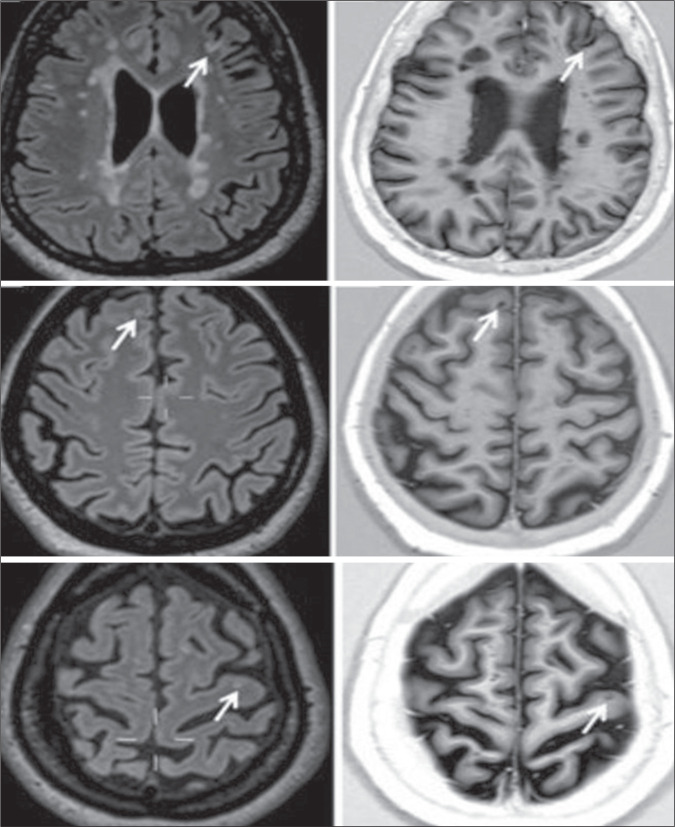
Axial 3D FLAIR (left) and axial PSIR (right): leukocortical (type I) lesion (top); juxtacortical lesion (middle); and intracortical (type II) lesion (bottom).

**Figure 2:**
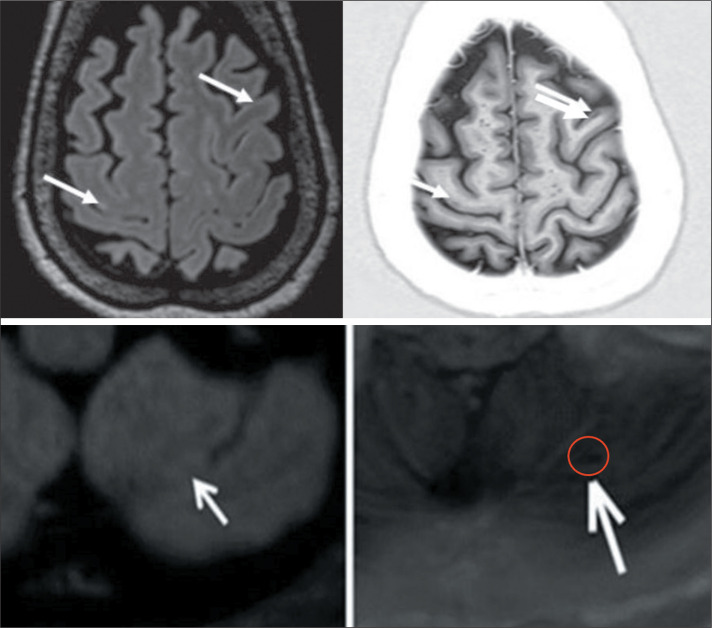
Lesions detected only on PSIR sequences. Axial 3D FLAIR (left) and axial PSIR (right): intracortical (type I) lesion (top) in the right precentral gyrus and left middle frontal gyrus; and infratentorial lesion (bottom).

The sample comprised 71 patients, all with the relapsing-remitting MS phenotype. The mean age was 47.3 ± 11.7 years. Among the 71 patients evaluated, 47 (62%) were female, the mean score on the EDSS was 2.4 ± 1.8, and the mean duration of disease was 12.9 ± 7.1 years. The raters detected 1,796 lesions on the FLAIR sequences (mean of 8.4 lesions/patient/rater), all of which were also visualized on the PSIR sequences (Figure 1). On the PSIR sequences, the raters detected 870 lesions (mean of 4.0 lesions/patient/rater) that were not visualized on the FLAIR sequences (Table 1), translating to approximately 50% additional lesions detected (Figure 2). Most (84%) of the lesions were in the supratentorial compartment.

**Table 1:** Frequency of cortical and juxtacortical lesions in patients with MS.

MRI sequence	Rater 1^*^ Mean ± SD^‡^	Rater 2^*^ Mean ± SD^‡^	Rater 3^†^ Mean ± SD^‡^
FLAIR	14.0 ± 14.2	7.7 ± 11.5	3.8 ± 4.5
PSIR	8.3 ± 8.0	2.7 ± 2.8	1.3 ± 1.3
PSIR *vs.* FLAIR (reclassification)	2.1 ± 2.2	1.7 ± 2.3	1.5 ± 1.9

* One year of experience.

† 14 years of experience.

‡ Counts per patient.

There were 28 patients (39.4%) in whom no cortical lesions were detected on the FLAIR sequences. In 12 of those patients, the raters found cortical lesions on PSIR images that were not apparent on FLAIR images. Therefore, the adjusted number of lesion-free individuals was 16 (22.5% of the sample). There was no significant difference between the patients without cortical lesions on FLAIR images and those without cortical lesions on PSIR images, in terms of mean age (46 ± 11.9 years *vs*. 43.6 ± 11.8 years; *p* = 0.47) or the mean of duration of disease (12.5 ± 6.8 years *vs.* 11.0 ± 6.3 years; *p* = 0.68). However, the mean EDSS score was 40% lower in the latter group (1.5 ± 0.9 *vs*. 2.5 ± 1.8).

The PSIR and FLAIR sequences both showed satisfactory interrater agreement in distinguishing between patients with and without lesions (mean agreement, 80.0%; κ_FR_, 0.58-0.62). For the reclassification of lesions, the mean agreement was 73.2%, with an κ_FR_ ranging from 0.56 to 0.58 (Table 2). The raters reclassified 366 lesions (1.7 lesions/patient/rater), corresponding to 20% of the lesions detected on the FLAIR sequences. The most common reclassification was from juxtacortical lesions to type I cortical lesions (in 81.5%), followed by juxtacortical lesions to type II cortical lesions (in 11.5%), juxtacortical lesions to white matter lesions (in 4.5%), and white matter lesions to juxtacortical lesions (in 2.5%).

**Table 2:** Interrater reliability for the detection and reclassification of cortical and juxtacortical lesions in patients with MS.

MRI sequence	Comparison	κ_FR_	95% CI	Agreement
FLAIR	R3 *vs.* R1	0.62	0.44-0.75	84.5%
	R3 *vs.* R2	0.60	0.42-0.73	87.3%
PSIR	R3 *vs.* R1	0.61	0.40-0.80	81.7%
	R3 *vs.* R2	0.58	0.40-0.71	76.2%
PSIR *vs.* FLAIR	R3 *vs.* R1	0.56	0.37-0.70	71.8%
(reclassification)	R3 *vs.* R2	0.58	0.40-0.71	74.6%

R3, rater 3; R1, rater 1; R2, rater 2.

The ICC demonstrated excellent interrater agreement for the number of lesions reclassified and satisfactory agreement for the total lesion counts (Table 3). Spearman’s ρ showed a significant correlation between the total number of lesions detected on PSIR sequences and the EDSS score (ρ = 0.35; *p* < 0.001). However, the total lesion count was not found to correlate significantly with any of the other variables assessed.

**Table 3:** Interrater reliability for the total counts of cortical and juxtacortical lesions in patients with MS.

MRI sequence	ICC	95% CI
FLAIR	0.73	0.49-0.85
PSIR	0.67	0.30-0.83
PSIR *vs.* FLAIR (reclassification)	0.91	0.87-0.94

**Figure 3:**
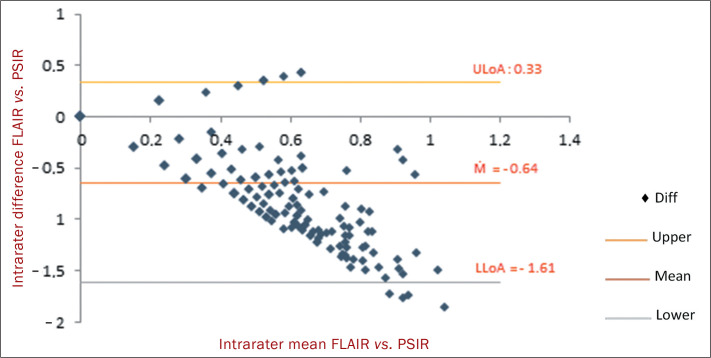
Intrarater differences and means of those differences. ULoA, upper limit of agreement; M, mean; LLoA, lower limit of agreement; Diff, difference.

As can be seen in the Bland-Altman plot (Figure 3), the mean intrarater difference between the FLAIR and PSIR sequence counts was 0.23 ± 3.09 lesions (inverse logit-transformed 95% CI: 0.19-0.27), approximately 97% of the differences remaining within the limits of the pre-established range for the expected agreement (Figure 3). The regression analysis ruled out a systematic variation in the means of differences (*p* = 0.27), thus excluding a proportion bias in the differences between the FLAIR and PSIR sequences.

## DISCUSSION

To our knowledge, this is the first study to assess the reliability of PSIR MRI among raters with markedly different levels of experience, which rules out the influence of training effects on the agreement coefficients^(21,23,24)^. We found clinically satisfactory levels of intrarater and interrater agreement for PSIR sequences, as well as showing that PSIR MRI had better sensitivity for the detection of cortical and juxtacortical lesions (50% better than FLAIR MRI), with no loss of reliability, and for the topographic reclassification of lesions.

The heterogeneity of the course of MS seems to be multifactorial, and it is likely that the topographic and volumetric characteristics of the demyelinating lesions contribute to that heterogeneity^(30-33)^. Therefore, MRI sequences that are more sensitive can help explain the variability in the course of the disease^(31-33)^. Despite having incorporated the presence of cortical lesions as a discriminating criterion for the spatial dissemination of MS, an expert panel concluded that conventional MRI sequences are incapable of accurately detecting and localizing cortical lesions and recommended the use of advanced sequences^(19)^. Likewise, the current consensus guidelines issued by the Magnetic Resonance Imaging in MS (MAGNIMS) group suggest considering the number and location of cortical lesions as a marker of MS progression, as long as the rater has a high level of expertise in MRI analysis^(32)^. Therefore, the accurate detection of cortical lesions has gained greater relevance, given its impact on the diagnosis and prognosis of MS. Consequently, assessing the reliability of advanced MRI sequences has become essential, and the present study meets that demand.

In our study, the raters detected higher numbers of lesions on PSIR images than on FLAIR images, with satisfactory interrater agreement for the detection and topographic reclassification of the lesions. In our study, the interrater ICC was moderate for the total lesion count on PSIR sequences, which is in contrast with the excellent agreement reported in some other studies^(16,17)^. However, in those studies, the raters had more experience than did raters 1 and 2 in our study. Overall, the higher agreement coefficients in previous studies suggest that training improves the rate of cortical lesion detection. However, our results indicate that a lack of training does not reduce the clinical efficacy of PSIR sequences.

The ICC showed excellent interrater agreement for the total number of lesions reclassified, whereas the κ_FR_ statistic showed only moderate interrater agreement for lesion reclassification. One possible explanation for this discrepancy is the difference in the size of the observed lesions. In the case of small lesions, the exact topographic localization of cortical lesions can be a challenge for less experienced examiners. However, the size of the lesions does not seem so relevant for their counting. That might have also accounted for the fact that the ICC for the lesion counts was higher on FLAIR sequences than on PSIR sequences. Because larger lesions are easier to visualize, they are easier to count, leading to higher agreement coefficients. The fact that the FLAIR and PSIR sequences presented good intrarater agreement and repeatability on the Bland-Altman plot, with mean differences close to zero, indicates a non-significant systematic bias between the methods. The CIs and SDs of the mean differences in agreement remained at values without clinical significance and within the parameters established *a priori*. The intrarater mean of differences ≤ 3.3 lesions (upper limit of agreement added to the SD, after inverse logit transformation) suggested the potential for interchangeability between the sequences.

Cortical lesions constitute a common finding in MS, and the frequency of such lesions in the present study was comparable to those reported in studies involving patients with MS phenotypes similar to those identified in our sample^(3,34-36)^. In our study, 77.5% of the patients presented cortical lesions, which is higher than the proportions reported in studies that included patients in the early stages of relapsing-remitting MS or clinically isolated syndrome, all of which were around 20%^(36,37)^. In contrast, studies including patients with progressive forms of MS showed higher proportions of patients with cortical lesions, ranging from 82% to 100%^(8,13,15,16)^. These findings suggest that the MS stage, which is directly associated with the EDSS score, as well as being indirectly associated with the duration of disease and with patient age, correlates with an increased risk of cortical lesions. We detected a significant correlation between the number of lesions detected on PSIR sequences and the EDSS scores, which is in line with data in the literature. A previous study, conducted by Nielsen et al.^(9)^, which used advanced 7.0-T MRI, found a significant association between cortical lesions and the EDSS score. A more recent study, conducted by Magliozzi et al.^(31)^, suggested that cortical pathology has a robust correlation with physical and cognitive disability in MS. These data should alert neuroradiologists to be aware of the clinical and demographic features of individual patients and to look for red flags during an MRI analysis.

In various studies, the presence of cortical lesions has been shown to be a robust independent predictor of the MS disability progression^(8,9,17,31,33,34,38,39)^. In the present study, the detection of cortical lesions on PSIR sequences (lesions that were not detected on FLAIR sequences) decreased the number of lesion-free patients by 43%. This reclassification lead to a relevant 40% reduction in the mean EDSS score when compared with the lesion-free group as determined on the FLAIR sequences. The reclassification from negative to positive for cortical lesions can have a significant influence on the prognosis of MS, given that the presence of cortical lesions has been correlated with pronounced disease activity and predisposition to progress to the advanced stages of the disease^(40)^. Our results indicate that the use of PSIR MRI is crucial for identifying patients with false-negative results for cortical lesions. Notably, 20% of the lesions detected on FLAIR sequences were topographically reclassified on PSIR sequences, and > 90% of the reclassifications were from juxtacortical to cortical. Juxtacortical lesions can occur in natural aging and may or may not be associated with progression of MS^(8,9,11,13,40)^. Therefore, if the presence of cortical lesions is considered a criterion for MS activity, their reclassification can influence decisions regarding treatment. One concern of the MAGNIMS group is the need for a higher level of expertise for the detection of cortical lesions in MRI analysis. The clinically satisfactory agreement observed in the present study, even among raters with limited experience, suggests that MRI protocols including PSIR sequences have an advantage over other MRI protocols, such as those including DIR sequences^(16-18)^. Although the MAGNIMS group recommended using the presence of cortical lesions as a marker of MS progression, they emphasized the low sensitivity of traditional MRI sequences for the detection of such lesions. However, our findings raise a question about using the emergence of cortical lesions as a definition of MS progression. One concern is that standardized advanced MRI sequences for the accurate detection of cortical lesions are not always available in some clinical settings.

Our study has some limitations. First, the fact that DIR sequences were not included in the local MRI protocol impeded the comparison with some data in the literature. In addition, we did not assess the cognitive status of the patients, which correlates significantly with cortical lesions in MS. However, we considered that evaluation to be beyond the scope of our study, the main objective of which was to assess the reliability of PSIR MRI, although such evaluation could be valuable in future studies of the validity of the PSIR protocol. Finally, the differences between PSIR sequences without volumetric acquisition and volumetric FLAIR sequences, in terms of spatial resolution and slice thickness (3.0 mm and 1.0 mm, respectively) should be taken into consideration. Those differences might even have influenced the detection and reclassification of some lesions, possibly leading to lower agreement coefficients, in our patient sample.

## CONCLUSION

Cortical and juxtacortical lesions have taken on greater relevance after being incorporated into the diagnostic criteria for MS. New, advanced MRI sequences, such as PSIR, with a higher sensitivity to detect such lesions, have been gaining importance in investigative protocols, which creates the need to assess their accuracy in the clinical context. Our results indicate that PSIR sequences perform better in the detection and topographic localization of cortical lesions than do conventional MRI sequences, such as FLAIR sequences. In addition, our data show that the detection of cortical lesions by PSIR MRI affects the EDSS scores, which could influence decisions regarding the treatment of MS. Future prospective studies aimed at determining whether and to what extent the findings on PSIR MRI correlate with physical and cognitive function over time in patients with MS could consolidate support for its incorporation into the routine MRI protocol for such patients.

## References

[1] Friese MA, Schattling B, Fugger L. (2014). Mechanisms of neurodegeneration and axonal dysfunction in
multiple sclerosis. Nat Rev Neurol.

[2] Dendrou CA, Fugger L, Friese MA. (2015). Immunopathology of multiple sclerosis. Nat Rev Immunol.

[3] Reich DS, Lucchinetti CF, Calabresi PA. (2018). Multiple sclerosis. N Engl J Med.

[4] Zuroff LR, Benjamins JA, Bar-Or A (2021). Inflammatory mechanisms underlying cortical injury in progressive
multiple sclerosis. Neuroimmunol Neuroinflamation.

[5] Walker CA, Huttner AJ, O’Connor KC. (2011). Cortical injury in multiple sclerosis; the role of the immune
system. BMC Neurol.

[6] Bouman PM, Steenwijk MD, Powels PJW (2020). Histopathology-validated recommendations for cortical lesion
imaging in multiple sclerosis. Brain.

[7] Beck ES, Gai N, Filippini S (2020). Inversion recovery susceptibility weighted imaging with enhanced
T2 weighting at 3T improves visualization of subpial cortical multiple
sclerosis lesions. Invest Radiol.

[8] Nelson F, Datta S, Garcia N (2011). Intracortical lesions by 3T magnetic resonance imaging and
correlation with cognitive impairment in multiple sclerosis. Mult Scler.

[9] Nielsen AS, Kinkel RP, Madigan N (2013). Contribution of cortical lesions subtypes at 7T MRI to physical
and cognitive performance in MS. Neurology.

[10] Farina G, Magliozzi R, Pitteri M (2017). Increased cortical lesion load and intrathecal inflammation is
associated with oligoclonal bands in multiple sclerosis patients: a combined
CSF and MRI study. J Neuroinflammation.

[11] Sethi V, Muhlert N, Ron M (2013). MS cortical lesions on DIR: not quite what they
seem?. PLoS One.

[12] Kuchling J, Paul F. (2020). Visualizing the central nervous system: imaging tools for
multiple sclerosis and neuromyelitis optica spectrum
disorders. Front Neurol.

[13] Filippi M, Preziosa P, Banwell BL (2019). Assessment of lesions on magnetic resonance imaging in multiple
sclerosis: practical guidelines. Brain.

[14] Favaretto A, Poggiali D, Lazzarotto A (2015). The parallel analysis of phase sensitive inversion recovery
(PSIR) and double inversion recovery (DIR) images significantly improves the
detection of cortical lesions in multiple sclerosis (MS) since clinical
onset. PLoS One.

[15] Nelson F, Poonawalla AH, Hou P (2007). Improved identification of intracortical lesions in multiple
sclerosis with phase-sensitive inversion recovery in combination with fast
double inversion recovery MR imaging. AJNR Am J Neuroradiol.

[16] Sethi V, Yousry TA, Muhlert N (2012). Improved detection of cortical MS lesions with phase-sensitive
inversion recovery MRI. J Neurol Neurosurg Psychiatry.

[17] Harel A, Ceccarelli A, Farrell C (2016). Phase-sensitive inversion-recovery MRI improves longitudinal
cortical lesion detection in progressive MS. PLoS One.

[18] Forslin Y, Bergendal A, Hashim F (2018). Detection of leukocortical lesions in multiple sclerosis and
their association with physical and cognitive impairment: a comparison of
conventional and synthetic phase-sensitive inversion recovery
MRI. AJNR Am J Neuroradiol.

[19] Thompson AJ, Banwell BL, Barkhof F (2018). Diagnosis of multiple sclerosis: 2017 revisions of the McDonald
criteria. Lancet Neurol.

[20] Cohen JF, Korevaar DA, Altman DG (2016). STARD 2015 guidelines for reporting diagnostic accuracy studies:
explanation and elaboration. BMJ Open.

[21] Kottner J, Audigé L, Brorson S (2011). Guidelines for reporting reliability and agreement studies
(GRRAS) were proposed. J Clin Epidemiol.

[22] Carpentier M, Combescure C, Merlini L (2017). Kappa statistic to measure agreement beyond chance in
free-response assessments. BMC Med Res Methodol.

[23] Fleiss JL, Levin B, Paik MC., Fleiss JL, Levin B, Paik MC. (2003). Statistical methods for rates and proportion.

[24] Sim J, Wright CC. (2005). The kappa statistic in reliability studies: use, interpretation,
and sample size requirements. Phys Ther.

[25] McGraw KO, Wong SP. (1996). Forming inferences about some intraclass correlations
coefficients. Psychological Methods.

[26] Bland JM, Altman DG. (1999). Measuring agreement in method comparison studies. Stat Methods Med Res.

[27] Calabrese M, Agosta F, Rinaldi F (2009). Cortical lesions and atrophy associated with cognitive impairment
in relapsing-remitting multiple sclerosis. Arch Neurol.

[28] Calabrese M, Rocca MA, Atzori M (2010). A 3-year magnetic resonance imaging study of cortical lesions in
relapse-onset multiple sclerosis. Ann Neurol.

[29] Mantha S, Roizen MF, Fleischer LA (2000). Comparing methods of clinical measurement: reporting standards
for Bland and Altman analysis. Anesth Analg.

[30] Filippi M, Rocca MA, Barkhof F (2012). Association between pathological and MRI findings in multiple
sclerosis. Lancet Neurol.

[31] Magliozzi R, Reynolds R, Calabrese M. (2018). MRI of cortical lesions and its use in studying their role in MS
pathogenesis and disease course. Brain Pathol.

[32] Wattjes MP, Cicarelli O, Reich DS (2021). 2021 MAGNIMS-CMSC-NAIMS consensus recommendations on the use of
MRI in patients with multiple sclerosis. Lancet Neurol.

[33] Lazeron RH, Langdon DW, Filippi M (2000). Neuropsychological impairment in multiple sclerosis patients: the
role of (juxta)cortical lesion on FLAIR. Mult Scler.

[34] Faizy TD, Thaler C, Ceyrowski T (2017). Reliability of cortical lesion detection on double inversion
recovery MRI applying the MAGNIMS-criteria in multiple sclerosis patients
within a 16-months period. PLoS One.

[35] Geisseler O, Pflugshaupt T, Bezzola I (2016). The relevance of cortical lesions in patients with multiple
sclerosis. BMC Neurol.

[36] Filippi M, Rocca MA, Calabrese M (2010). Intracortical lesions: relevance for new MRI diagnostic criteria
for multiple sclerosis. Neurology.

[37] Kolber P, Montag S, Fleischer V (2015). Identification of cortical lesions using DIR and FLAIR in early
stages of multiple sclerosis. J Neurol.

[38] Louapre C, Govindarajan ST, Giannì C (2016). The association between intraand juxta-cortical pathology and
cognitive impairment in multiple sclerosis by quantitative T2* mapping at 7
T MRI. Neuroimage: Clin.

[39] Calabrese M, Rinaldi F, Grossi P (2011). Cortical pathology and cognitive impairment in multiple
sclerosis. Expert Rev Neurother.

[40] Scalfari A, Romualdi C, Nicholas RS (2018). The cortical damage, early relapses, and onset of the progressive
phase in multiple sclerosis. Neurology.

